# Experimental Study on the Flexural Behavior of Lap-Spliced Ultra-High-Performance Fiber-Reinforced Concrete Beams

**DOI:** 10.3390/polym14112138

**Published:** 2022-05-24

**Authors:** Baek-Il Bae, Hyun-Ki Choi

**Affiliations:** 1 Department of Digital Architectural and Urban Engineering, Hanyang Cyber University, Seoul 04763, Korea; bibae@hycu.ac.kr; 2 Department Fire and Disaster Prevention Engineering, Kyungnam University, Changwon 51767, Korea

**Keywords:** UHPC, lap splice, flexural strength

## Abstract

In this paper, the structural performance of lap-spliced beams were investigated by the testing of 18-UHPFRC beams with and without a lap-spliced region and steel fiber. All test specimens were subjected to flexural load by using the four-point bending test. It was shown that test steel fiber inclusion in the ultra-high-strength matrix significantly increased the strength of the lap-spliced beams. The maximum strength of the specimen increased linearly with the increase in steel fiber volume fraction. The hybrid-type UHPFRC, with lower steel fiber volume fraction, using two types of steel fiber was effective in improving the bond strength. In order to verify the safety of lap splice length design methods by current code provisions and UHPFRC design recommendations, the average bond stress at the ultimate state was used to calculate the lap splice length, which makes reinforcement yielding, and this value, compared with design lap splice length. Most of the design equations were under the estimated bond stress of UHPFRC. However, the AFGC recommendation, which considers the tensile strength of UHPFRC, overestimates the bond stress of UHPFRC because this recommendation was developed from the direct pull-out test results.

## 1. Introduction

Ultra-high-performance fiber-reinforced concrete (UHPFRC) has now been widely investigated by many government and university research institutes based on the development of fiber-reinforced concrete (FRC) and reactive powder concrete (RPC). UHPFRC has remarkable strength under compression and tension, and ductility under tension. These mechanical advantages make UHPFRC members thin and a long span bridge can be constructed with reduced self-weight. Using UHPFRC, the rebar details of reinforced concrete members can be changed more simply because of its performance improvement [1,2,3,4,5,6,7].

Recently developed commercial UHPFRC needs steam curing [8,9]. For this purpose, most UHPFRC is usually used as precast members. Therefore, the bond between UHPFRC, rebars, and splice length becomes a very important design consideration. Tepfers [10] reported that the stress applied to the concrete by the reinforcing bars occurs radially with respect to the perimeter of the reinforcing bars, and that splitting cracks occur along the longitudinal direction of the reinforcing bars due to the corresponding stresses. Normal strength concrete usually has much lower tensile strength and many design code provisions neglect the tensile strength of normal strength concrete. In order to prevent the splitting failure of normal strength concrete members, reinforcement is arranged in the transverse direction of bonded or spliced rebars [11,12,13]. In UHPFRC, it is possible to resist the stress radiated in the circumferential direction of the reinforcing bar due to the high tensile resistance capability of the UHPFRC matrix; thus, it is a new alternative in the development and splice length design. Therefore, design recommendations of development and splice length design need to be derived considering the mechanical advantage of UHPFRC. However, research regarding bond, development, and splice design for UHPFRC members has been very limited [14].

The easiest way to define the bond properties between reinforcing bars and concrete is the direct pull-out test. The direct pull-out test between the UHPFRC and the reinforcement is continuing from the early stage of UHPFRC research to the present. Holschemacher et al. [15] conducted a direct pull-out test on reinforcing bars with a diameter of 10 mm for a total of five UHPFRC mixes. Experiments were carried out with the standard method of RILEM [16] and modified specimens to induce splitting tensile failure. As a result, the maximum compressive strength of UHPC was 148 MPa and the ultimate bond stress was almost 70 MPa. According to Jungwirth’s study [17], the ultimate bond stress of UHPFRC was found to be a maximum of 66 MPa, and the magnitude of the ultimate bond stress varied depending on the bond length and the diameter of the rebar. Leutbecher [18] also reported the magnitude of the ultimate bond stress through direct pull-out tests and reported that the magnitude of the ultimate bond stress varies with the size of the concrete cover and the volume fraction of steel fibers. Significantly, it was found that steel fiber was effective in increasing the ultimate bond stress when splitting tensile failure occurs due to small concrete cover depth. Saleem et al. [19]. conducted a direct pull-out test with a 10 mm and 22 mm diameter reinforcement with a UHPFRC cover depth of 13 mm. The magnitude of the ultimate bond stress varied with the bond length and the diameter of the rebar. It was confirmed that an ultimate bond stress of 17 MPa was exhibited. Bae et al. [20]. conducted a direct pull-out test with UHPFRC compressive strength, volume fraction of steel fiber, and concrete cover depth and reported that ultimate bond strength of up to 30 MPa occurred.

As a result of previous studies on the direct pull-out test, there was a difference according to the variables. However, when UHPFRC is used, it can have a high ultimate bond stress between 30 and 70 MPa. However, as described in the report of CI 408 [21], the bond stress capacity can be overestimated due to the constraint by the compressive stress distributed in the concrete when the direct pull-out test is performed. Unlike the direct pull-out test, there is no section where the concrete around the rebar under tension receives compressive force. The design of lap splice length through the direct pull-out test may not be safe due to the overestimation of bond strength. Therefore, the safety evaluation and design method of the lap splice length is recommended to use the test method in which the concrete around the rebar under tensile stress does not have compressive force or less. ACI408 proposes development and a splice length calculation formula through the bending test of lap-spliced beams [21].

The UHPFRC member is also often subjected to tensile stress only rather than subjected to compressive stress. Therefore, it is necessary to study the lap-spliced UHPFRC member. However, unlike ordinary concrete, the study about lap splice length was very limited in UHPFRC flexural members. A study of the behavior of the lap-spliced beam was performed by Lee [22]. The main experimental variables were the amount of steel fiber and the lap splice length. As a result of the bending strength tests of the lap-spliced beam, the behavior with the appropriate displacement ductility ratio was obtained when the lap length of 10d_b, which is 10 times the diameter of the lap-spliced rebar, which was not included the steel fiber. This phenomenon was similar in UHPFRC beams with 1% fiber content. In the case of the beam with 2% volume fraction of steel fiber, it had a similar displacement ductility ratio to the control specimen, which had no lap splice region. On the other hand, it was reported that premature fracture occurs when the lap splice length of 5d_b. Kim et al. [23] conducted a direct pull-out test and bending test to investigate the bond strength of UHPFRC. The main variables were concrete cover and lap splice length. Experimental results showed that UHPC can exhibit adequate strength even with a very short joint length of 2.2 times the rebar diameter. Until now, the authors have found that there was no experimental test program for a UHPFRC lap-spliced beam test.

As recommended in ACI 408 [21], design code provisions regarding bond behavior between concrete and rebar recommended being determined through a bending test of the lap-spliced beams. Significantly, in the previous experimental study on UHPFRC flexural members [12,23], it was found that the required lap-spliced length that yielded the tensile rebar was much shorter than that of the existing design criteria [12,13] for normal strength concrete. It will be necessary to review it. Therefore, in this study, the load resistance of UHPFRC flexural members with short lap splice length was evaluated experimentally.

## 2. Design Recommendations for Lap Splice Length

For the design of lap splice lengths of members using UHPFRC, a review of currently applicable design criteria has been carried out. Design recommendations recommended by AFGC [24] and KICT [25] can be applicable to UHPFRC members. As a result of reviewing the relevant design recommendations, the basic form of the lap splice length design formula was based on the design criteria for normal concrete members. Therefore, this study also examined the design criteria for normal concrete members. AFGC [24] was based on Eurocode 2 [13], and KICT [25] was based on KCI [12].

### 2.1. Eurocode 2

In Eurocode2 [13], the following Equation (1) was used to estimate the lap splice length under tension. For the required lap splice length shown in Equation (2), the shape of bars, concrete cover, confinement by transverse reinforcement and pressure, and percentage of reinforcement lapped in length are considered.

(1) $$l_{0}=\alpha_{1}\alpha_{2}\alpha_{3}\alpha_{5}\alpha_{6}l_{b,rqd}\geq l_{0,min}$$

(2) $$l_{b,rqd}=\left(\phi /4\right)\left(\sigma_{sd}/f_{bd}\right)$$

where, $\alpha_{1}$ is coefficient considering the shape of bars and 1.0 can be used for straight bars. $\alpha_{2}$ is coefficient for concrete cover. For straight bar, $\alpha_{2}=1-0.15\left(c_{d}-\phi \right)/\phi$. This value cannot be smaller than 0.7 and cannot be greater than 1.0. $\alpha_{3}$ is the coefficient about transverse reinforcement $\alpha_{3}=1-K$. K is the coefficient about the location of transverse reinforcement and longitudinal reinforcement. λ is the lap-spliced rebar ratio about whole tensile reinforcement and calculated by using equation $\lambda =\left(\sum A_{st}-\sum A_{st,min}\right)/A_{s}$, where $A_{st}$ is the sectional area of transverse reinforcement in the region of splice and development length of the longitudinal rebar. $\sum A_{st,min}$ is the minimum sectional area of transverse reinforcement and calculated by equation of $\sum A_{st,min}=1.0A_{s}\left(\sigma_{sd}/f_{yd}\right)$. $A_{s}\;$ is the area of the lap-spliced bar. $\alpha_{5}$ is the lateral pressure which confines the longitudinal reinforcements. $\alpha_{6}$ can be calculated by using equation ${\left(\rho_{1}/25\right)}^{0.5}$ but not exceeding 1.5 nor less than 1.0 where $\rho_{1}$ is the percentage of reinforcement lapped within $0.65l_{0}$ from the center of the lap length considered. ϕ is the diameter of the lap-spliced rebar, $\sigma_{sd}$ is the design stress of the bar at the position from where the anchorage is measured from, and $f_{bd}$ is the ultimate bond stress which can be calculated by Equation (3).

(3) $$f_{bd}=2.25\eta_{1}\eta_{2}f_{ctd}$$

$f_{ctd}$ is the design value of the concrete tensile strength, $\eta_{1}$ is a coefficient related to the quality of the bond condition and the position of the bar during concreting, and $\eta_{2}$ is a coefficient related to the bar diameter. However, in EC2, the $f_{ctd}$ value is limited to the $f_{ctd}$ value of C60/55 in order to consider the brittle fracture tendency of high-strength concrete.

$l_{0,min}$ is the minimum required lap splice length and calculated by Equation (4).

(4) $$l_{0,min}>max\left\{0.3\alpha_{6}l_{b,rqd};15\phi ;200\;mm\right\}$$

### 2.2. AFGC Recommendation

The AFGC Recommendation [24], which is the design recommendation of the structure that reflects the performance of ultra-high-performance fiber-reinforced concrete, basically uses the same lap splice length calculation method as EC2 [13]. However, in EC2, since the compressive strength level of concrete is limited with respect to the ultimate bond strength, the application of UHPFRC is difficult. Therefore, the ultimate bond strength formulas are adjusted in the corresponding design recommendations. In addition, the minimum lap splice length was modified so that the required bond length, which is shortened by the increase in the bond strength, can be reflected in the structural design. The ultimate bond stress between the UHPFRC and the rebar can be determined from Equation (5), and the minimum lap splice length can be determined from Equation (6).

(5) $$f_{bd}={\eta \eta }_{1}\eta_{2}\kappa f_{ctk,el}/\gamma_{c}$$

where η is the coefficient about the types of reinforcements, such as steel rebars and prestressing tendons. For ribbed steel reinforcement, η is 2.25, which is also used in Eurocode2. κ can be calculated by using $1+0.5f_{ctfm}/f_{ctm,el}$ and this value cannot be greater than 1.5. where $f_{ctfm}$ is the mean maximal post-cracking stress and $f_{ctm,el}$ is the mean stress of limit of elasticity under tension.

(6) $$l_{0,min}>max\left\{0.3\alpha_{6}l_{b,rqd};\delta 15\phi ;\delta 200\;mm\right\}$$

where $\delta =1-0.5f_{ctfm}/f_{ctm,el}\;$ and cannot be smaller than 0.5.

### 2.3. KCI2012

In KCI2012 [12], two types of classes are used for the calculation of lap-spliced length, Class A and B, according to the stress level of the reinforcing bar, the types of members, and the amount of reinforcement. For reinforcing bars exceeding D35, lap slice is not applicable. The straight development length of the steel rebar, $l_{d}$, can be calculated from Equation (7). Class A and B are classified by the following. For Class A splice, the area of reinforcement provided is at least twice that required by analysis over the entire length of the splice and the spliced reinforcement within the required lap length is not more than 1/2 of the total reinforcement. For all other cases except for Class A, the coefficient for Class B can be used. The lap splice length is calculated as 1.0$l_{d}$ for Class A joints and 1.3$l_{d}$ for Class B joints. The minimum lap splice length is limited to 300 mm.

(7) $$l_{d}=\frac{0.90d_{b}f_{y}}{\lambda \sqrt{f_{ck}}}\frac{\alpha \beta \gamma }{\left(\frac{c+K_{tr}}{d_{b}}\right)}$$

The value $\left(c+K_{tr}\right)/d_{b}$ shall not be greater than 2.5. α is the position factor of bars arrangement, 1.3 for top reinforcing bars (horizontal bars cast by fresh concrete in order to make the development length or under-bonded lap joint greater than 300 mm) and 1.0 for other cases. Β is the coated factor of bars and 1.0 can be used for deformed uncoated bars. γ is the size factor of the reinforcing bar. 0.8 can be used for bars not greater than D19, and 1.0 can be used for other cases. c is the minimum concrete cover. $K_{tr}$ is the transverse reinforcement index, which can be calculated by $40A_{tr}/\left(sn\right)$. $A_{tr}$ is the cross-sectional area of transverse reinforcement. s is the maximum center-to-center spacing of transverse reinforcement, within development length. n is number of bars being spliced or developed along the plane of splitting.

### 2.4. KICT

KICT uses the same method of KCI for the calculation of lap splice length but uses different coefficients. The straight line length, $l_{d}$, for the design of UHPFRC can be obtained by multiplying the basic fixation length, $l_{db}$, by the correction coefficient. If transverse reinforcement bars are not located in development or lap-spliced region, multiply the basic development length by 1.2α for rebars with diameters less than or equal to D19, and multiply by 1.5α for rebars with D22 or greater diameters. α is the coefficient considering the location of rebar, 1.3 can be used for the top bar, and 1.0 can be used for other cases. The minimum lap splice length is recommended to 100 mm and basic development length, $l_{db}$, can be calculated by using Equation (8).

(8) $$l_{db}=\frac{0.186d_{b}f_{yk}}{\phi_{m}\sqrt{f_{ck}}}$$

where $d_{b}$ is the diameter of the rebar, $f_{yk}$ is the characteristic yield strength of the rebar, and $\phi_{m}$ is material reduction factor.

## 3. Test Program

### 3.1. Subsection Variables and Specimen Detail

**Figure 1:**
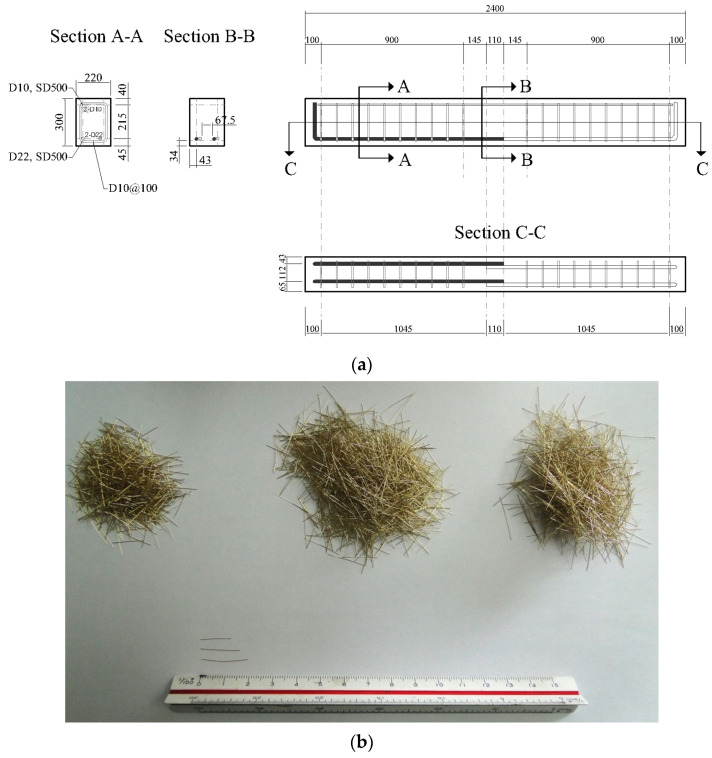
Details of test specimen (Unit: mm) and material: (**a**) Details of test specimens; (**b**) Fiber used for construction of test specimens.

**Figure 2:**
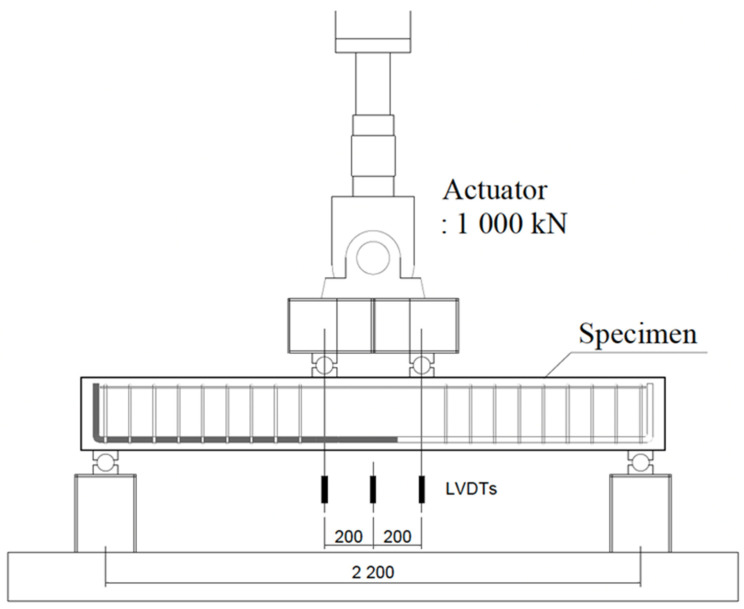
Test Setup.

In this study, the 4-point bending test of the lap spliced beams was carried out to investigate the effectiveness of lap splice on the flexural strength development according to the use of UHPFRC as the concrete matrix. The characteristics of UHPFRC for the performance of lap-spliced beams were investigated by setting the compressive strength of concrete, the length of the lap-spliced region, the amount of steel fiber, and the type of steel fiber as the main variables. The compressive strength design of the concrete was set to 120 MPa and 180 MPa. The design yield strength of the reinforcing bar was set to 500 MPa. According to the previous study on the lap splice length of UHPFRC [22], when a lap splice length of 10$d_{b}$ was used, the yield strength of the bar with was 400 MPa. In addition, according to the commentary of the KICT design recommendation for UHPFRC, reinforcement with a development length of 2.2$d_{b}$ can be yielded. Therefore, in this study, test specimens with splice lengths of 10$d_{b}$ and 5$d_{b}$ were tested. Because higher yield strength of reinforcement was used, the test specimen with a splice length of 15$d_{b}$ was tested. In order to investigate the bond performance of UHPFRC in the lap splice region, the volume fraction of micro steel fiber was set to 1 and 2%. In addition, the effect of different types of steel fiber reinforcing methods on the flexural behavior of lap-spliced UHPFRC beams was investigated by applying a hybrid fiber, which was composed of two different types of steel fiber. The diameter of tensile reinforcement was 22.2 mm so that the lap splice length reducing the effect of smaller bar size [12] was not considered. Figure 1 shows the detail of one of the lap spliced beam specimens and used steel fibers. Table 1 summarizes the details of the test specimens. In order to induce the complete flexural failure of the specimen, shear reinforcement was placed within the shear span. The loading points of all the test specimens were set to be the same and the lap splice region was located between the loading points. MTS actuators with a capacity of 1000 kN were used for the loading. The test setup is shown in Figure 2.

### 3.2. Material Properties

The main purpose of this study is to evaluate the lap splice performance for UHPFRC flexural members. According to Tepfers [10] and Goto [26], bond failure between the rebar and concrete is determined by the tensile resistance of concrete. Therefore, the bond strength of UHPFRC to reinforcing bars with greatly increased tensile strength will be greatly improved, and short lap splice lengths can be applied to UHPFRC members. Therefore, the tensile performance of UHPFRC was investigated in this study. The main factor determining the tensile strength of UHPFRC is the amount and type of steel fiber mixed into UHPFRC. Therefore, in this study, a study was conducted on specimens with and without steel fiber. A total of six UHPFRC mixes were used, and the mix proportion of each mix is summarized in Table 2. The mix proportions used in this study were based on RPC and were produced with low water-to-binder ratios in both the 120 and 180 series. In order to solve the problem of low flowability, which was caused by the low water-to-binder ratio and the inclusion of steel fiber, a high range water reducing agent was used for all the mix proportions. A straight-type and 0.2 mm diameter steel fiber was used. Because the effect of the stress transfer between cracks after crack initiation differs depending on the length of the steel fiber [27], a different length of the steel fiber was used. Three lengths of steel fiber were used, 13 mm, 16 mm, and 19 mm. Steel fibers 13 mm in length were used in a 1% and 2% volume fraction in a single inclusion. 16 mm and 19 mm steel fibers were mixed with a 1% and 0.5% volume fraction, respectively, and used as 1.5% hybrid fibers. The tensile strength of the steel fiber used was 2600 MPa and the modulus of elasticity was 200,000 MPa.

**Figure 3:**
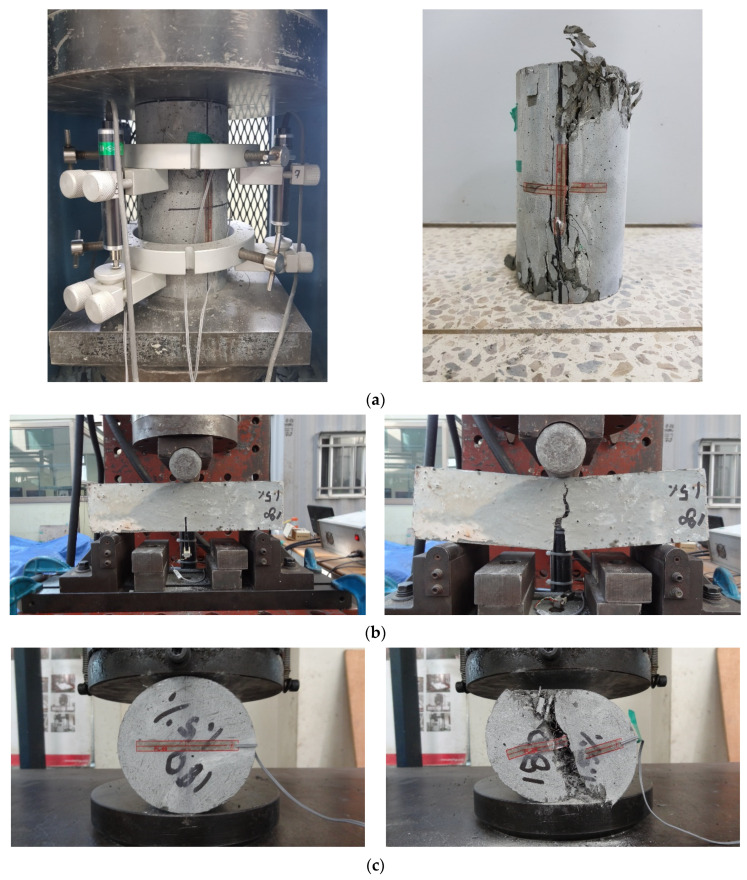

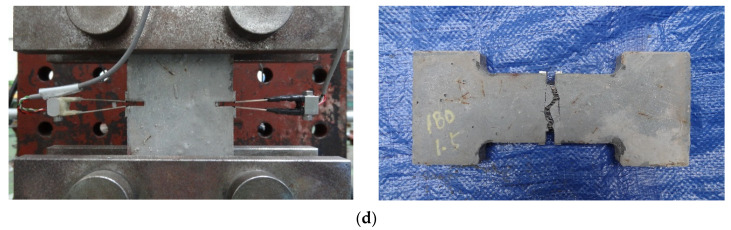
Material test setup and typical failure of material test specimens: (**a**) Compressive strength test; (**b**) Flexural strength test; (**c**) Splitting strength test; (**d**) Direct tensile strength test.

**Figure 4:**
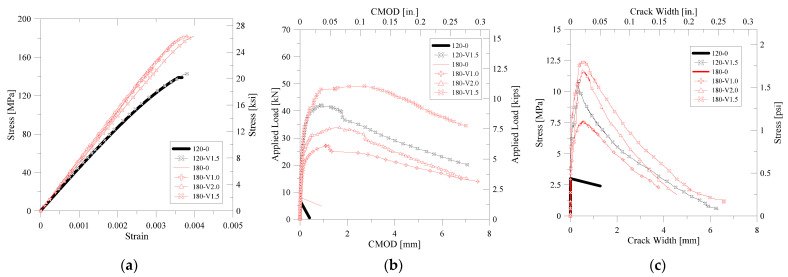
Material test results: (**a**) Compressive stress–strain relation; (**b**) Applied flexural load-CMOD relation; (**c**) Direct tensile stress–crack width relation.

As a result of the analysis of existing studies and design criteria, the design of lap splice length has been greatly influenced by the strength of material, such as the compressive strength of concrete and yield strength of the reinforcing bar. For the detailed examination of material strength, compressive strength, flexural tensile strength, splitting tensile strength, and direct tensile strength, tests were performed for all mix proportions used in this study. Test setups and failure modes are illustrated in Figure 3. The mechanical properties of each were measured by KS 2405 [28], JCI [29], KS 2423 [30], and KICT [25]. Table 3 summarizes the test results. In order to characterize the UHPFRC member, it is necessary to define the characteristics under UHPFRC under compressive stress and tensile stress. In this study, three tensile test methods were used as well as a compressive test. The results of the flexural tensile strength and direct tensile strength tests were obtained by stress-CMOD (Crack Mouth Opening Displacement). Compression, flexural, and direct tensile test results are shown in Figure 4a–c, respectively.

The reinforcing bar used in this study was the SD500 based on the KS standard [31] with a yield strength of 500 MPa. D22 reinforcing bars were used as the main steel bars and D10 was used as the compression bars and transverse steel bars. The mechanical properties of reinforcing bars used in the test specimens are as follows. The yield strength and tensile strength of D10 were measured at 541 MPa and 653 MPa, respectively, and the yield strain was 0.0027. The yield strength and tensile strength of D22 were 530 MPa and 681 MPa, respectively, and yield strain was 0.00265. The nominal diameter of D22 used as a reinforced bar in this study was 22.2 mm, the cross section was 387.1 mm^2^, and the perimeter was 70 mm. The average distance between ribs was 15.5 mm and the rib height was 1.1 mm. Test results are shown in Table 4.

### 3.3. Measurement Plan

As the lap splice length applied to the test specimens was considerably shorter than the lap splice lengths used in ordinary concrete, the strain of the reinforcing bars itself could not be examined in order not to affect the bond strength between the reinforcement and UHPFRC. In addition, UHPFRC could affect the resistance value of the strain gauge by performing high temperature steam curing. Therefore, in this study, strain gauges were not attached directly to the reinforcing bars to examine the strain at the reinforcing bars, and strain gauges were attached to the compression side to indirectly examine the stresses acting on the reinforcing bars.

## 4. Test Results

### 4.1. Mode of Failure

**Figure 5:**
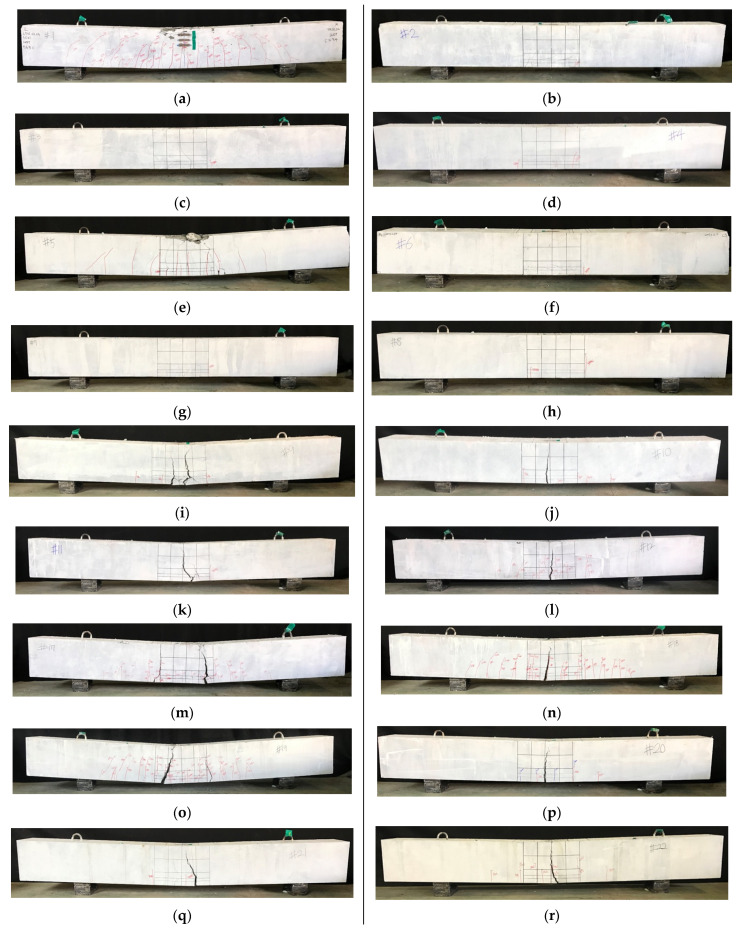
Failure of test specimens: (**a**) 120-0-0; (**b**) 120-15db-0; (**c**) 120-10db-0; (**d**) 120-5db-0; (**e**) 180-0-0; (**f**) 180-15db-0; (**g**) 180-10db-0; (**h**) 180-5db-0; (**i**) 120-10db-V1.5; (**j**) 120-5db-V1.5; (**k**) 180-10db-V2.0; (**l**) 180-5db-V2.0; (**m**) 120-0-V1.5; (**n**) 180-0-V2.0; (**o**) 180-0-V1.5; (**p**) 180-5db-V1.5; (**q**) 180-10db-V1.0; (**r**) 180-5db-V1.0.

All specimens were designed to fail in flexural mode when no lap splices were present, resulting in failure due to flexural failure. However, in the case of the test specimen lap splice, the maximum flexural strength was not experienced and the load carrying capacity was lost. Figure 5 shows the final failure conditions of all specimens.

120-0-0 and 180-0-0, 120-0-V1.5, 180-0-V2.0 and 180-0-V1.5, which did not have a lap spliced region, all failed due to the crushing of concrete at compression fiber after the yielding of tensile reinforcements. The initial cracks occurred in the vertical direction from the lower end to the upper end of the loading point. After the continuous cracking occurred between the loading points, the crushing of concrete occurred at the extreme compression fiber, which are shown in Figure 5. The reinforcement effect of the steel fiber was found in the distribution of cracks. 180-0-V2.0, compared to 180-0-0, shows a large number of cracks dispersed, and 180-0-V1.5 using hybrid fibers showed more cracks than 180-V2.0. However, when reinforced with steel fiber, the final fracture appeared after the concentration of strain due to crack localization. When the ultra-high-strength concrete matrix was used, the fracture patterns of the lap-spliced region was splitting failure.

**Figure 6:**
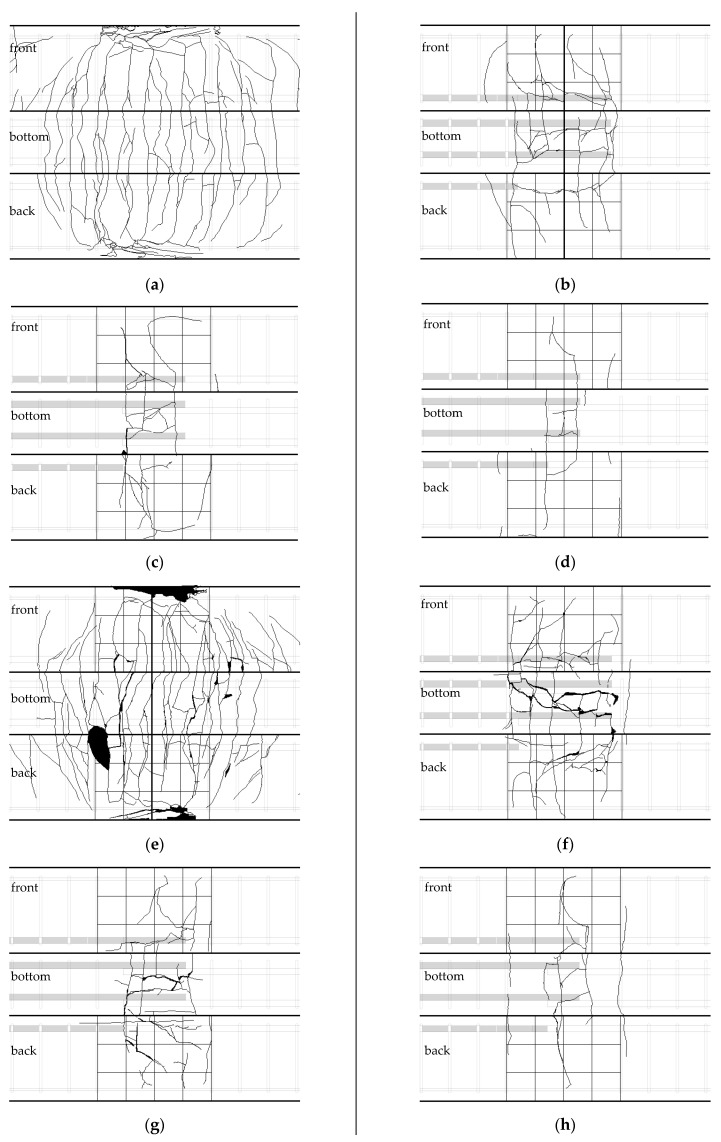

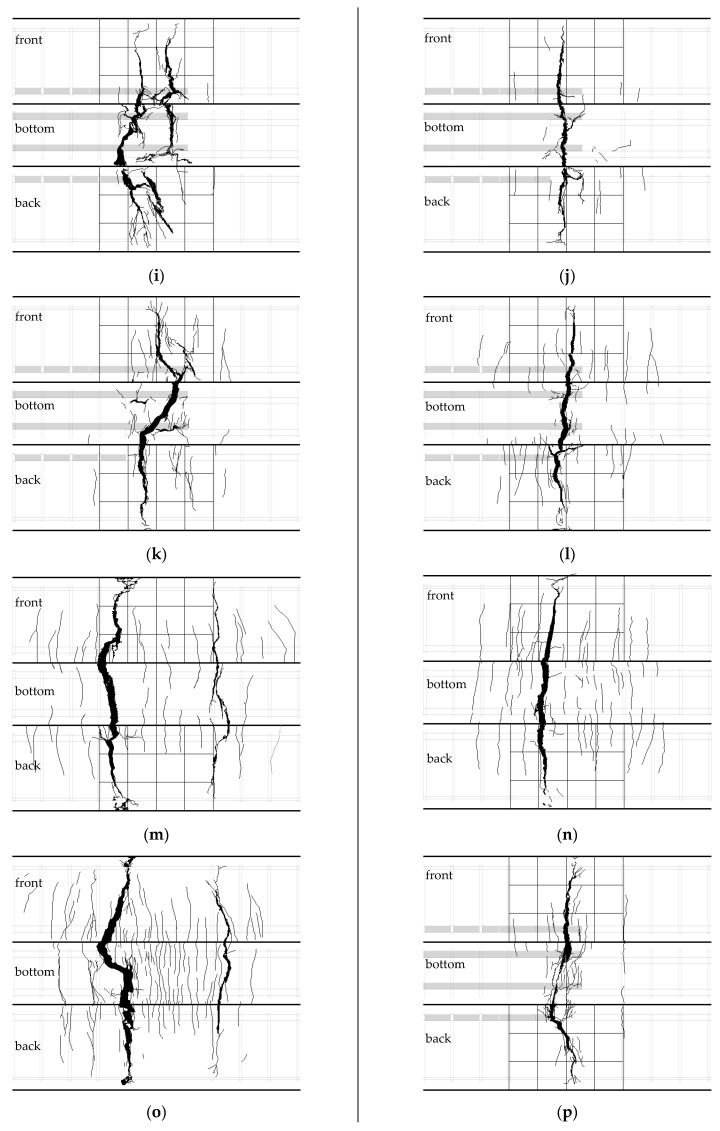

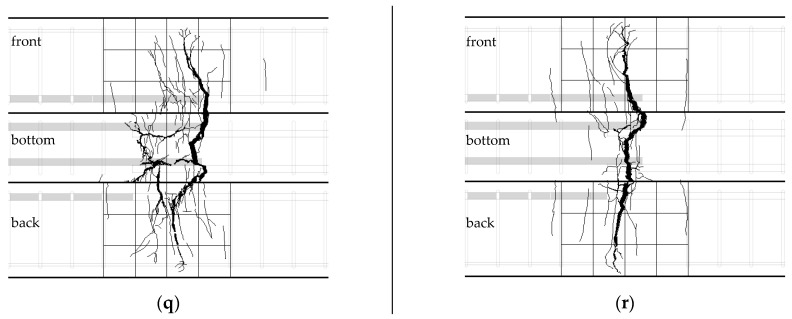
Cracking aspect of test specimens at lap-spliced region: (**a**) 120-0-0; (**b**) 120-15db-0; (**c**) 120-10db-0; (**d**) 120-5db-0; (**e**) 180-0-0; (**f**) 180-15db-0; (**g**) 180-10db-0; (**h**) 180-5db-0; (**i**) 120-10db-V1.5; (**j**) 120-5db-V1.5; (**k**) 180-10db-V2.0; (**l**) 180-5db-V2.0; (**m**) 120-0-V1.5; (**n**) 180-0-V2.0; (**o**) 180-0-V1.5; (**p**) 180-5db-V1.5; (**q**) 180-10db-V1.0; (**r**) 180-5db-V1.0.

Figure 6 shows the final fracture pattern of the lower surface of the specimen in the lap-spliced region. In the case of the test specimens with a splice length of 15$d_{b}$, the longitudinal cracks occurred in the lower part of the test specimens after the occurrence of the vertical flexural cracks. At the same time, the cracks occurred in the diagonal direction from the side to the tensile steel bars. The similar failure pattern occurred for test specimens with a 10$d_{b}$ splice length. The specimens with a lap splice length of 5$d_{b}$ also failed in a similar manner. However, vertical cracks and diagonal cracks did not occur in the lap splice region. According to the difference in the compressive strength of concrete, the crack inclination angle was higher when concrete of 180 MPa was used.

In order to investigate the reinforcement effect of steel fiber on the bond between reinforcing steel and concrete, experiments were carried out on specimens with steel fibers in 10$d_{b}$ and 5$d_{b}$ lap splice length specimens. Hybrid fiber inclusion test specimens 120-10db-V1.5 and 120-db-V1.5 did not experience the abrupt loss of load bearing capacity and cracking, unlike 120-10db-0 and 120-5db-0. The cracks generated in the longitudinal direction of the reinforcing bars spread along the vertical direction of the longitudinal direction of the reinforcing bars. At the same time, the width of the flexural cracks widened and fracturing progressed. However, in the 10$d_{b}$ specimen, the amount of crack diffusion was larger than that of the 5$d_{b}$ specimen.

The fracture behavior of the specimens reinforced with 1% and 2% microfibers in the specimen 180-10db-0 was similar to that of the 120 series reinforced with steel fiber. As the amount of steel fiber was increased, the spreading area of cracks was widened, and it was confirmed that crack localization was more likely to occur rather than the widening of the cracks due to the blowout of reinforcing bars. It is confirmed that when a 1 and 2% volume fraction of microfibers are applied to 180-5db-0, the specimens were separated by the large flexural crack at the center of the end of the spliced rebar. However, it was confirmed that the number of flexural cracks diffused when a 2% volume fraction of steel fiber applied was larger. In addition, the splitting cracks on the lower side and the splitting cracks on the side were not observed, and it was confirmed that the beams were separated from the ends of the lap-spliced rebar. It was found that hybrid fibers included in the 180-5db-0 test specimen had similar behavior to those with 180-5db-V1.0 and V2.0. However, it was confirmed that the number of longitudinal cracks at the lower surface of the beam was greatly reduced.

### 4.2. Load-Deflection Curves

**Figure 7:**
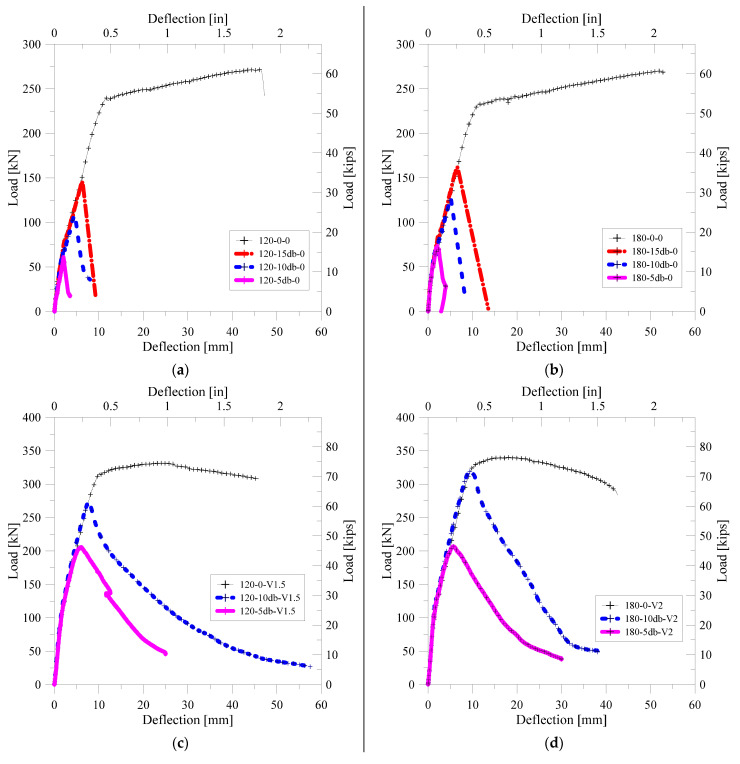

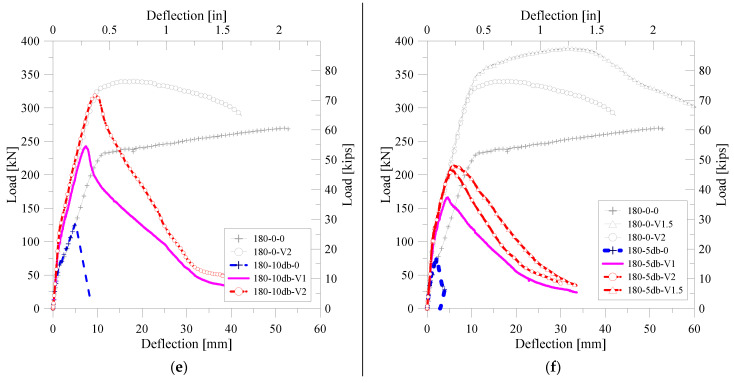
Load-deflection relation of tested specimens: (**a**) Specimens with 120-0 matrix; (**b**) Specimens with 180-0 matrix; (**c**) Specimens with 120-V1.5 matrix; (**d**) Specimens with 180-V2.0 matrix; (**e**) Specimens with 10 db lap length; (**f**) Specimens with 5 db lap length–effect of steel fiber.

Reinforced concrete members with lap splice should behave in the same way as beams without lap splice and should have adequate ductility. In order to evaluate the flexural performance of the lap-spliced UHPFRC, the load-deflection relationship of each specimen is shown in Figure 7. In addition, the yield strength and maximum strength of each specimen are summarized together with the deflection at the strength. In order to investigate the lap splice performance on the flexural strength of the ultra-high-strength concrete with no steel fiber, and load-deflection curves for the specimens without steel fiber are shown in Figure 7a,b. It was confirmed that 120-0-0 and 180-0-0, which have no lap splice region, had high strength and ductility. However, it could be seen that all of the lap-spliced specimens did not reach the yield strength of 120-0-0 and 180-0-0. Lap-spliced specimens with no steel fiber lost their load bearing capacity immediately after the maximum strength of each specimen.

Figure 7d,e shows the load-deflection curves for lap-spliced test specimens with steel fiber. It was confirmed that the load bearing capacity of the specimen reinforced with hybrid steel fibers in the matrix with a compressive strength of 120 MPa was gradually decreased, unlike the abrupt fracture tendency of the specimen not reinforced with the steel fiber. It showed higher flexural strength than the specimens not reinforced with steel fiber. Specimens with the matrix of 180-V2.0 showed similar behavior to those with the 120-V1.5 matrix. It was confirmed that the maximum strength of the specimen had a 10$d_{b}$ lap splice length close to the yield strength of the specimen without lap splice. However, the loss of load carrying capacity was found to be larger in the case of the specimen with the 10$d_{b}$ lap splice length.

In order to examine the change in the structural performance of beams with a 10$d_{b}$ lap-spliced length according to the types and amount of steel fiber, load-deflection curves for specimens with 180-0, 180-V1.0, and 180-V2.0 were constructed, as shown in Figure 7e. It could be confirmed that the flexural stiffness and flexural strength were greatly increased with the increase in the volume fraction of the steel fiber. Significantly, specimens with a 10$d_{b}$ lap splice length have shown the yield strength of non-spliced specimens. As shown in Figure 7f, it was confirmed that the increase in the lap splice performance increased with the increase in steel fiber volume fraction similar to that of the 10$d_{b}$ specimens. However, these specimens cannot reach the yield strength of the test specimen without lap splice. The test specimen with the hybrid-type steel fiber had slightly higher flexural strength than that of the microfiber-reinforced specimens. The decrease-tendency of load bearing capacity was slower than that of the specimen reinforced with microfiber.

## 5. Applied Stress to Tensile Reinforcements

### 5.1. Strain of Steel at Lap-Spliced Region

**Figure 8:**
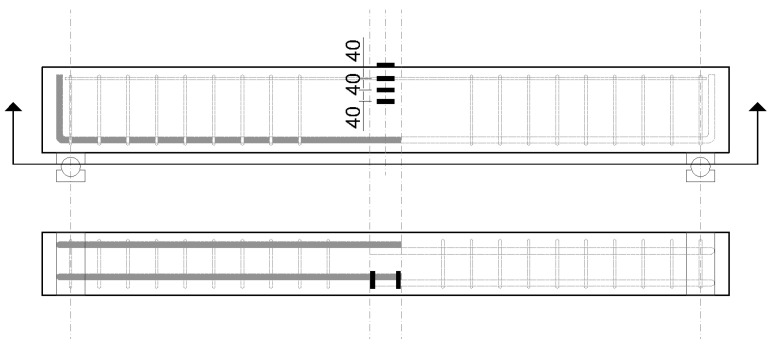
Strain measurement location.

**Figure 9:**
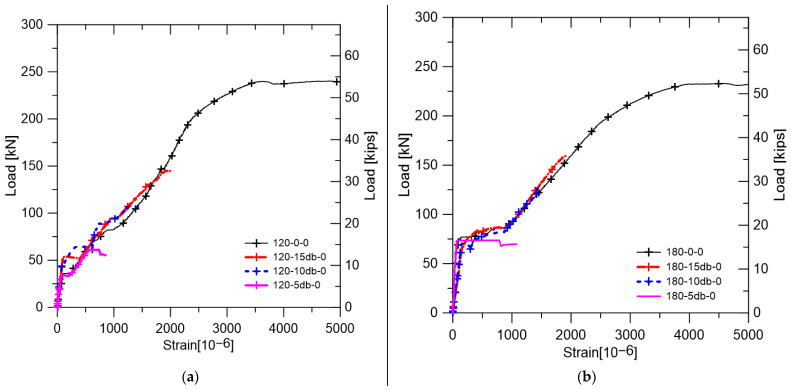

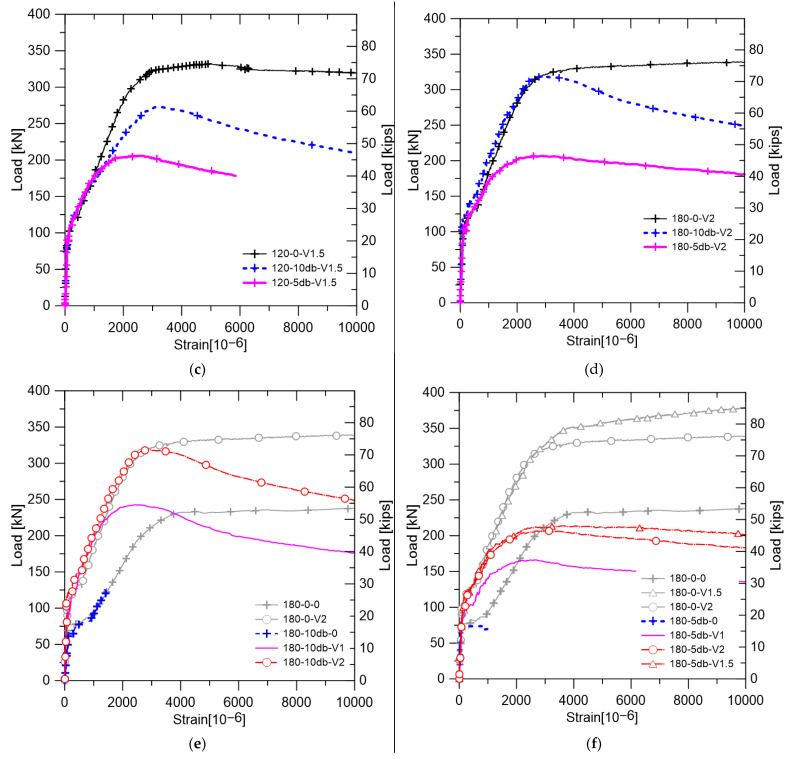
Strain at steel reinforcement location: (**a**) Specimens with 120-0 matrix; (**b**) Specimens with 180-0 matrix; (**c**) Specimens with 120-V1.5 matrix; (**d**) Specimens with 180-V2.0 matrix; (**e**) Specimens with 10 db lap length; (**f**) Specimens with 5 db lap length effect of steel fiber.

The strain at the rebar location was calculated by the extrapolation of the strain obtained from the concrete strain gauges installed at the compression side of each specimen as shown in Figure 8. The change in strain at the position of the reinforcing bars of each specimen is shown in Figure 9. Figure 9a,b showed the strain of specimens not reinforced with steel fiber. The magnitude of the strain at maximum load was found to be larger as the lap splice length was longer. In Figure 9c,d, the effect of lap splice length on the strain of lap-spliced bars was examined. Unlike Figure 9a,b, the steel strain of the rebar did not abruptly drop but gradually increased and decreased before and after experiencing load carrying capacity. Significantly, the 10$d_{b}$ specimen showed the yield strain of the reinforcing bar unlike specimens without steel fiber. However, the 5$d_{b}$ lap-spliced test specimens could not experience the yielding of reinforcement.

The load–strain relationships for specimens with a lap splice length of 10$d_{b}$ are shown in Figure 9e. When the steel fiber was not reinforced, the strain of the reinforcing bar when experiencing the maximum load was smaller than that of the specimen with no lap splice. When the microfiber with 2% volume fraction was applied, it was confirmed that fracture occurred just before the yielding of the steel rebar. In this case, the steel strain at maximum strength was not significantly different from each other.

Figure 9f showed the load–strain relation of test specimens with a short lap splice length 5$d_{b}$. It was confirmed that the maximum strength of unreinforced test specimen was shown adjacent to the initial cracking load of test specimen without lap splice. On the other hand, test specimen with 1% microfibers had shown the significant increase of strain at maximum load. Test specimens with hybrid fiber and 2% volume fraction microfiber had similar strain at maximum strength. The gradual decrease in the load carrying capacity of the hybrid fiber-reinforced test specimen was shown rather than the 2% volume fraction micro fiber-reinforced test specimen.

According to Figure 9, it was confirmed that the strain of test specimens without steel fiber have shown similar trends of specimens without lap splice before experiencing maximum strength. On the other hand, steel fiber-reinforced lap-spliced specimens have shown a similar tendency in strain variation before experiencing the 70 to 80% of maximum strength. After that, rapid increase in strain was shown. In particular, this phenomenon could be seen more clearly in the case of the specimens having the short splice length of 5$d_{b}$. This was considered to be the phenomenon in which the splitting cracks occurring in the lap splice region were confined by the steel fiber. Stress at the tensile rebar was calculated based on the strain measurements and are summarized in Table 5.

### 5.2. Strain Calculated by Sectional Analysis

In this study, since the stress acting on the reinforcing bar was not directly measured, the strain at the reinforcing bar position was examined through the sectional analysis as well as the extrapolation using the concrete gauges. In order to investigate the flexural behavior of reinforced concrete members, the following assumptions were applied and the methodology used in previous studies was applied [32].

(1) plane sections before bending remain plane after bending;
(2) steel rebar perfectly bonded to the concrete under compression and tension;
(3) the tensile stress–strain relation should be considered for the UHPFRC matrix but the tensile stress–strain relation can be neglected when concrete is not reinforced with steel fiber.

**Figure 10:**
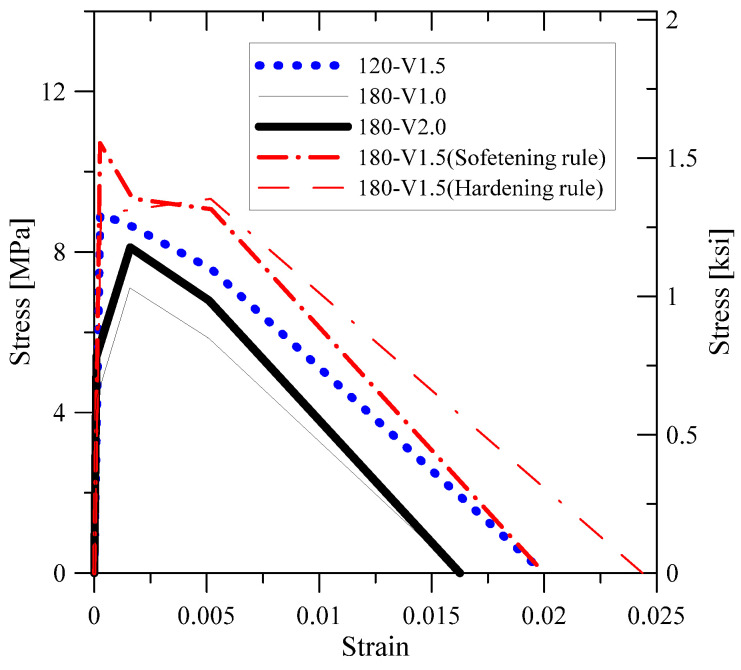
Tensile stress-strain relation of tested matrix.

The stress–strain relation of UHPFRC should be defined. In this study, for this purpose, tensile strength tests were conducted. Because UHPFRC have significantly large displacement capacity, CMOD was used as the displacement value. In order to apply the stress–CMOD relation to sectional analysis, it was necessary to transfer the stress–CMOD relation to the stress–strain relation. AFGC [24] and JCI [29] recommended the method to transfer the stress–CMOD relation to the stress–strain relation. In this study, the AFGC recommendation was used to construct the stress–strain relation of UHPFRC under tension. The tensile stress–strain relationship of UHPFRC is shown in Figure 10. For the 120-V1.5 and 180-V1.5 matrixes which were reinforced with hybrid steel fiber, a strain-hardening model was used. For the matrixes with microfiber, a strain-softening model was applied.

**Figure 11:**
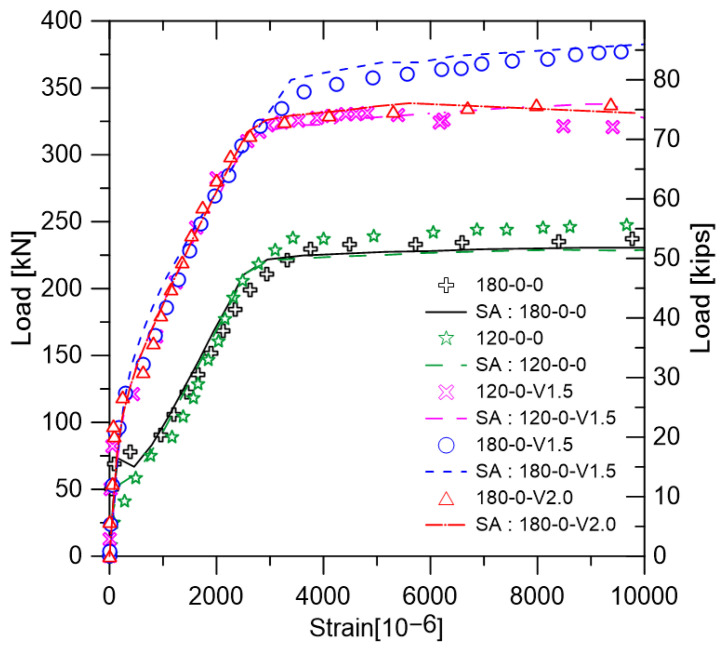
Sectional analysis results.

Figure 11 shows the load–steel strain relation calculated by the sectional analysis. Test specimens without lap splice were analyzed by using sectional analysis. In the case of 180-0-V1.0, as shown in Figure 11, the experiment was not conducted. However, as shown in Figure 11, sectional analysis conducted in this study had high accuracy. As a result of the comparison between the analysis results and the experimental results, it was confirmed that it could be used to predict the behavior of 180-5db-V1.0 and 180-10db-V1.0. The stresses acting on the reinforcing bars under the maximum load state of each specimen are summarized, as shown in Table 5.

### 5.3. Expansion of Concrete Adjacent to Lap-Spliced Rebar

When examining the ultimate bond stresses through lap splice or bond test, the ultimate bond stress can be calculated by using Equation (9), assuming the bond stress was evenly distributed over the lap-spliced or bond region.

(9) $$u_{c}=\frac{T}{l_{d}\left(\pi d_{b}\right)}=\frac{A_{b}f_{s}}{l_{d}\left(\pi d_{b}\right)}=\frac{d_{b}f_{s}}{4l_{d}}$$

where $u_{c}$ is the average ultimate bond stress, T is the tensile force applied to the reinforcements, $l_{d}$ the is lap-spliced or bond length, $d_{b}$ is the diameter of reinforcement, $A_{b}$ is the cross-sectional area of reinforcement, $f_{s}$ is the applied stress to reinforcement. Generally, T is calculated through stress at the time of maximum strength development.

**Figure 12:**
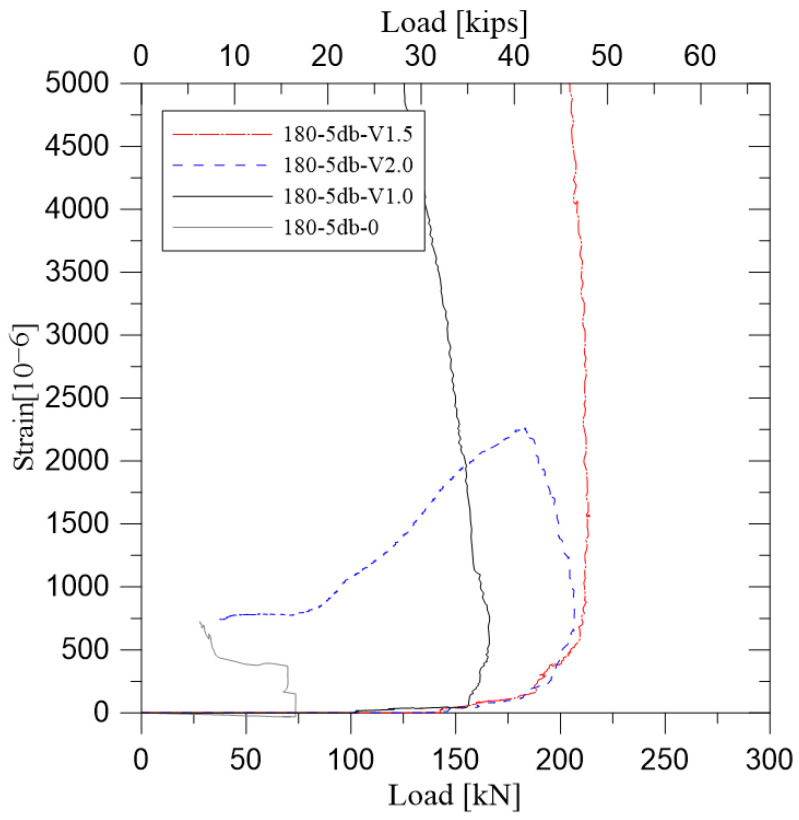
Expansion of concrete adjacent to the tensile lap-spliced reinforcements.

As shown in Figure 9a,b, there was no difference of strain at failure between lap-spliced test specimens and test specimens without lap splice, in the case of non-fiber-reinforced test specimens, because of the abrupt failure experiencing maximum load. However, according to the remaining figures in Figure 9, it could be seen that specimens with a lap splice length of 5$d_{b}$ exhibited greater strain than those of specimens which were not lap-spliced at maximum strength. This is caused by the dispersion of the cracks, which can be seen in Figure 6. When the steel fiber was not reinforced, fracture progresses at the same time as cracking occurs. However, it was considered that the specimen reinforced with steel fiber was caused by the bridging effect of the steel fiber, causing the crack to open slowly and causing deformation of the larger reinforcing bars. This could be confirmed from the measurement results of the strain gauge attached to the beam bottom as shown in Figure 8. As shown in Figure 12, specimens reinforced with steel fiber, unlike 180-5db-0, which was not reinforced with steel fiber, showed a low rate of increase in strain before longitudinal cracking but a sudden increase in strain after specific load. In this study, the stress at the beginning of the crack was determined from the measured values, and the magnitude of the applied stress was calculated. The stress determined through investigating lateral-direction strain gauges are summarized in Table 5.

## 6. Discussion

### 6.1. Ultimate Average Bond Stress

**Figure 13:**
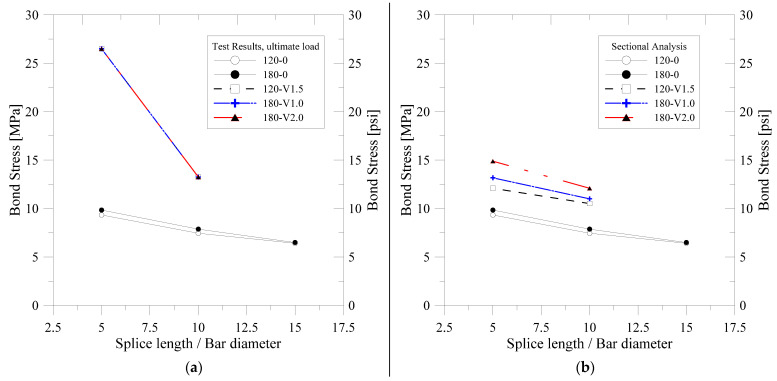

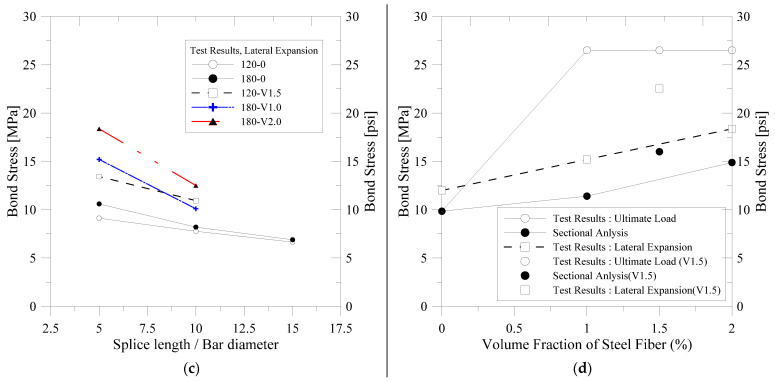
Average bond stress at lap-spliced region: (**a**) Bond stress calculated based on the ultimate load of test specimens; (**b**) Bond stress calculated based on the sectional analysis results; (**c**) Bond stress calculated based on the lateral expansion of bottom of the test specimen; (**d**) Bond stress–steel fiber volume fraction relation.

Figure 13 shows the magnitude of the bond stress calculated by the experiment of each specimen according to the change in lap splice length and steel fiber content. Figure 13a–c show the maximum bond stresses calculated by the maximum load of test results and the maximum bond stresses derived from the cross-section analysis and the bond stresses at crack occurrence, respectively. The ultimate bond stress decreased with the increase in lap splice length as shown in all the figures of Figure 9.

As shown in Figure 13a, the ultimate average bond stresses of the specimens not reinforced with steel fiber decreased with increasing lap splice length. The change in the compressive strength of concrete did not significantly influence to the ultimate average bond stress.

All specimens reinforced with steel fiber were found to experience yielding of the rebar. However, it was difficult to confirm that the lap splice was perfect because the lap-spliced specimens did not reach the flexural strength of the specimens without lap splice. Therefore, it was considered that the stress will be overestimated due to the bond stress determined by the stress of the reinforcing bar at the maximum strength of test specimens. Figure 13c shows the bond stresses based on the stresses of the reinforcing bars at the time of crack extension in the transversal direction of the reinforcing bars. These bond stresses were higher than bond stresses determined from the sectional analysis, as shown in Figure 13b. It was considered that this phenomenon was caused by the increase in strength until the softening phenomenon occurs after the initial cracking as shown by the tensile stress–strain relationship of UHPFRC. Since the section analysis results are more conservative than the measurement results, it was considered that the safety of UHPFRC members with lap splice could be secured designs using the results of section analysis.

Figure 13d shows the change in the bond stresses according to the change in steel fiber volume fraction. All investigated specimens had lap splice lengths of 5 db. As shown in Figure 13a–c, the steel fiber-reinforced specimens experienced yielding at maximum strength, so that the increase and decrease in bond stress with the variation in steel fiber volume fraction could not be investigated. However, it was confirmed that the bond stress increases linearly with the increase in the amount of steel fiber reinforcement investigating the results of sectional analysis and measurement results considering lateral expansion of the bottom of the beams. Therefore, it can be concluded that the increase in the tensile strength with the increase in the steel fiber content directly affects the increase in the average bond stress. However, when hybrid fibers were used, the bridging effect after cracking occurred more efficiently than when using single microfibers with a higher volume fraction, and therefore, the tendency to increase the bond stress according to the amount of reinforcing steel fiber was significantly different. It was confirmed that the strength of the test specimens using the hybrid fiber was increased more effectively than that of the case of using only the microfibers.

### 6.2. Required Lap Splice Length

Table 6 summarizes the bond stresses related to each specimen. Based on the calculated bond stress, the lap splice length required to induce yielding of the rebar is calculated and shown in Table 6. According to Table 6, even when the same matrix was used, the bond stress varied depending on the lap splice length. Azizinamini [33] reported, from the experimental results of the lap-spliced high-strength concrete members, that the stress concentration at loading point could appear. UHPFRC members were reinforced with steel fiber, so stress dispersion may occur along the lap-spliced region. However, because much higher compressive strength was provided to UHPFRC, it was shown that stress concentration did not disappear despite being reinforced with steel fiber.

For the safe design of the lap splice length of UHPFRC members, based on the design criteria of the two normal concrete structures examined in this study and the design recommendation of UHPFRC, the required lap splice length was calculated and is summarized in Table 6. In the case of KCI [12], KICT [25], and EC2 [13], it was confirmed that the lap splice length was determined according to the level of concrete compressive strength because the inclusion of steel fiber was not considered in the calculation of lap splice length. As the design recommendations of AFGC [24] considered the tensile strength of the UHPFRC to the lap splice length, the required lap splice length for the yielding of reinforcement had been changed according to the steel fiber content. Furthermore, in the case of AFGC [24], the design of the minimum lap splice length required was found because the very short required lap splice length was calculated when considering the tensile strength of UHPFRC.

For the specimens not reinforced with steel fiber, it was found to be conservative, except for the required lap splice length of KICT [25] for a compressive strength of 180 MPa. KICT [25] is a design recommendation for UHPFRC, but it was impossible to design considering the change of steel fiber because this design recommendation was developed from only one mix proportion. In the case of steel fiber inclusion, all design criteria or recommendations, except AFGC [24], led to conservative design results. It was shown that UHPFRC lap splice performance was overestimated for all specimens when applying the AFGC [24] design recommendation. This was because the ultimate bond stress between UHPFRC and reinforcing bars was overestimated and the design formula of AFGC, until recently, was derived based on the direct pull-out test.

## 7. Conclusions

For the safe design of UHPFRC structural members, the effect of matrix compressive strength, type of steel fiber, and mixing amount on the flexural behavior of lap-spliced UHPFRC beams was evaluated experimentally. For this purpose, the flexural load was applied to the lap-spliced beam specimens with a concrete cover less than 2$d_{b}$, and the following conclusions were obtained:

(1) As a result of evaluating the flexural performance of the ultra-high-performance concrete using the concrete matrix of 120 MPa and 180 MPa, it was confirmed that compressive strength does not have a great influence on flexural strength. However, the increase in flexural strength due to the inclusion of the steel fiber was confirmed, and the hybrid-type steel fiber with the two lengths of steel fiber had higher bending strength. Ductility decreased with the inclusion of steel fiber because of the increase in tensile force of the section and crack localization;
(2) The lap-spliced beams, which were not reinforced with steel fiber, failed due to splitting failure similar to ordinary concrete lap-spliced beams. As a result of experiments at 5 times, 10 times, and 15 times of the diameter of tensile reinforcement, it was found that the fracture occurred due to the cracks at the bottom surface in the longitudinal direction of the reinforcing bars in both lap splice beams with the compressive strengths of 120 and 180 MPa;
(3) In the case of UHPFRC lap spliced beams, initial cracking was delayed and did not experience abrupt loss of load carrying capacity after experiencing maximum load due to the dispersion of cracks. For all the lap-spliced test specimens, the maximum strength did not reach the flexural strength, but the rebar experienced yielding at maximum strength;
(4) It could be confirmed that the strength of lap-spliced UHPFRC test specimens changed with the inclusion of steel fiber. The maximum strength of the specimen increased linearly with the increase in the steel fiber volume fraction. The hybrid-type UHPFRC with lower steel fiber volume fraction using two types of steel fiber was effective in improving the bond strength;
(5) As a result of the measurement of the expansion strain of the concrete around the reinforced concrete, it was confirmed that when the steel fiber is not reinforced, the maximum strength and the strain increase at the same time. On the other hand, when reinforced with steel fiber, the maximum strength was not reached even when the rapid expansion strain started. It can be seen that the steel fiber prevents the pullout failure bridging the longitudinal cracks;
(6) As a result of evaluating the lap splice performance with the average bond stress, it was found that the hybrid fiber reinforced test specimen had the highest strength. Although the matrix types are the same, the average bond stress varies depending on the lap splice length. Therefore, the bond stress concentration phenomenon and the nonlinear distribution phenomenon are also observed in UHPFRC;
(7) As a result of the sectional analysis, it was confirmed that the magnitude of the average bond stress was smaller than that of the experimental results, and it was considered that the result of the sectional analysis through the stress–strain relation proposed by AFGC can be used in designing the lap splice length of UHPFRC flexural members;
(8) It was confirmed that the splice length was required to be conservative when designing the UHPFRC member as the current design standard. The KICT design recommendation is conservative because it is a design recommendation derived from only one type of UHPFRC matrix. The AFGC design recommendation considered the tensile strength of UHPFRC in the determination of the bond stress, but it was found that the bond stress was overestimated as the bond stress calculation formula was derived by the direct pull-out test. Therefore, in order to construct a safe design formula, additional research based on the lap-spliced beam test, which is more disadvantageous in estimating the bond stress, should be performed.

## Figures and Tables

**Table 1:** Details of test specimens.

ID	b	d	$l_{s}$	$d_{b}$	$l_{s}/d_{b}$	c	$f_{ck}$	$f_{y}$	$f_{y,tr}$	$d_{b,tr}$	$V_{f}$	$L_{f}$	$D_{f}$
	(mm)					(mm)	(MPa)			(mm)	(%)	(mm)	
120-0-0	220	255	-	22.2 (D22)	-	-	120	500	400	9.5 (D10)	0	-	-
120-15db-0			330		15	43							
120-10db-0			220		10								
120-5db-0			110		5								
180-0-0			-		-	-	180						
180-15db-0			330		15	43							
180-10db-0			220		10								
180-5db-0			110		5								
120-10db-V1.5			220		10		120				1.5	16/19	0.2
120-5db-V1.5			110		5								
180-10db-V2.0			220		10		180				2	13	0.2
180-5db-V2.0			110		5								
120-0-V1.5			- - -		-	-	120						
180-0-V2.0							180						
180-0-V1.5											1.5	16/19	
180-5db-V1.5			110		5	43							
180-10db-V1.0			220		10						1	13	0.2
180-5db-V1.0			110		5								

$l_{s}$: lap splice length, $d_{b}$: diameter of tension reinforcements, c: minimum concrete cover based on ACI318-14, $f_{ck}$: compressive strength of concrete, $f_{y}$: yield strength of longitudinal steel, $f_{y,tr}$: yield strength of transverse steel reinforcements, $d_{b,TR}$: diameter of transverse reinforcements, $V_{f}$: volume fraction of steel fiber, $L_{f}$: length of steel fiber, $D_{f}$: diameter of steel fiber.

**Table 2:** Mix proportion of UHPFRCs.

ID	Water	Cement	Basalt Furnace	Silica Fume	Filler	Sand	Shrinkage Reducing Agent	Super Plasticizer	Steel Fiber (13/0.2)	Steel Fiber (19.5/0.2)	Steel Fiber (16.3/0.2)
	Unit Weight (kg/m^3^)										
120-0	204	781.5	136.8	58.6	234.4	859.6	7.8	18			
120-V1.5	204	781.5	136.8	58.6	234.4	859.6	7.8	18		78	39
180-0	170	799.5		199.9	239.9	879.5	8	18.4			
180-V1.0	170	799.5		199.9	239.9	879.5	8	18.4	78		
180-V2.0	170	799.5		199.9	239.9	879.5	8	18.4	156		
180-V1.5	170	799.5		199.9	239.9	879.5	8	18.4		78	39

**Table 3:** Mechanical characteristics of used UHPFRCs.

ID	*V_f_*	*E_c_*	*f_c_′*	*f_r_*	*f_sp_*	*f_t_*
	(%)	(MPa)				
120-0	0	44,302	140.8(1.90 *)		5.2(2.27 *)	2.4(0.25 *)
120-V1.5	2.0	44,767	142.2(4.26 *)	12.6(3.47 *)	15.9(1.60 *)	10.6(1.25 *)
180-0	0	52,046	173.3(5.59 *)		9.5(1.40 *)	3.0(0.13 *)
180-V1.0	1.0	51,253	174.0(4.37 *)	9.8(2.38 *)	12.43(1.22 *)	7.6(1.30 *)
180-V2.0	2.0	53,336	178.5(5.35 *)	13.2(2.88 *)	18.0(3.84 *)	11.6(2.25 *)
180-V1.5	1.5	52,333	175.9(5.56 *)	14.3(2.970 *)	20.1(3.77 *)	12.4(1.47 *)

*V_f_*: Volume fraction of steel fiber, *E_c_*: Elastic modulus of UHPC, *f_c_′*: compressive strength of cylinder, *f_r_*: tensile strength according to the back analysis notched specimen flexural test, *f_sp_*: splitting tensile strength of UHPC, *f_t_*: tensile strength according to the direct tension test, *: standard deviation.

**Table 4:** Mechanical characteristics of used rebars.

ID	*E_s_*	*f_y_*	*f_u_*	$\varepsilon_{y}$
	[MPa]			
D10	200,000	541	653	0.00270
D22	200,000	530	681	0.00265

*E_s_*: Elastic modulus of rebar (assumed), *f_y_*: yield strength of rebar, *f_u_*: tensile strength of rebar, $\varepsilon_{y}$: yielded strain of rebar.

**Table 5:** Stress at lap-spliced region measured and calculated values.

ID	*f_s,test,ult_*	*f* *_s,calc_*	*f_s,test,cr_*	*u_s,test,ult_*	*u* *_s,calc_*	*u_s,test,cr_*
	(MPa)					
120-15db-0	381	381	395	6.41	6.41	6.65
120-10db-0	295	295	308	7.44	7.44	7.77
120-5db-0	185	185	181	9.33	9.33	9.12
180-15db-0	386	386	409	6.49	6.49	6.89
180-10db-0	312	312	325	7.87	7.87	8.20
180-5db-0	195	195	210	9.84	9.84	10.60
120-10db-V1.5	530	417	434	13.25	10.51	10.94
120-5db-V1.5	530	240	266	26.50	12.09	13.43
180-10db-V2.0	530	479	495	13.25	12.09	12.50
180-5db-V2.0	530	295	364	26.50	14.89	18.37
180-5db-V1.5	530	317	447	26.50	16.00	22.54
180-10db-V1.0	530	358	400	13.25	9.03	10.10
180-5db-V1.0	530	226	301	26.50	11.4	15.20

*f_s,test,ult_*: ultimate tensile stress of lap-spliced reinforcements test results, *f_s,calc_*: ultimate tensile stress of lap-spliced reinforcements analysis results, *f_s,test,cr_*: longitudinal cracking tensile stress at lap-spliced region test results, *u_s,test,ult_*: ultimate bond stress test results, *u_s,calc_*: ultimate bond stress analysis results, *u_s,test,cr_*: longitudinal cracking bond stress test results.

**Table 6:** Required lap splice length.

ID	*l_d,rqd_*	*l_d,ACI_*	*l_d,KICT_*	*l_d,EC2_*	*l_d,AFGC_*	*l_d,specimen_*
	(mm)					
120-15db-0	442	612	390	932	715	330
120-10db-0	379					220
120-5db-0	322					110
180-15db-0	427	500	318	870	572	330
180-10db-0	359					220
180-5db-0	278					110
120-10db-V1.5	269	612	390	932	200(116)	220
120-5db-V1.5	219					110
180-10db-V2.0	235	500	318	870	181(102)	220
180-5db-V2.0	160					110
180-5db-V1.5	130				181(95)	110
180-10db-V1.0	291				196(160)	110
180-5db-V1.0	194					110

*l_d,rqd_*: required lap-spliced length calculated by using bond stress calculated by test results, *l_d,ACI_*, *l_d,KICT_*, *l_d,EC2_*, *l_d,AFGC_*: lap-spliced length calculated by using code provisions (KCI, KICT, EC2, AFGC), *l_d,specimen_*: lap-spliced length of test specimens.

## Data Availability

Not applicable.
